# Cannabidiol Decreases Metalloproteinase Activity and Normalizes Angiogenesis Factor Expression in UVB-Irradiated Keratinocytes from Psoriatic Patients

**DOI:** 10.1155/2021/7624389

**Published:** 2021-10-13

**Authors:** Agnieszka Gęgotek, Sinemyiz Atalay, Adam Wroński, Agnieszka Markowska, Elżbieta Skrzydlewska

**Affiliations:** ^1^Department of Analytical Chemistry, Medical University of Bialystok, Poland; ^2^Dermatological Specialized Center “DERMAL” NZOZ in Bialystok, Poland

## Abstract

The development of psoriasis is associated with the consequences of oxidative stress and inflammation leading to metabolic changes locally, in the skin cells, and systemically, in the blood. Therefore, the aim of this study was to analyze the effect of psoriasis vulgaris (PsV) and psoriatic arthritis (PsA) on the basal plasma/keratinocyte levels of matrix metalloproteinases (MMPs), tissue inhibitors of matrix metalloproteinases (TIMPs), and angiogenesis factors, as well as to evaluate the effect of CBD on these parameters in keratinocytes isolated from psoriatic/healthy individuals with and without in vitro irradiation by UVB. A quantitative chemiluminescent method of detection based on an ELISA protocol and zymography technique was used during analysis. It was shown that activity levels of MMP-9 and TIMP-2 in PsA plasma were higher than in PsV. Changes in the proteolytic activity were accompanied by an increase in markers of angiogenesis (angiopoietin-2, HGF, VEGF, TNF*α*, PDGF, FGF), where in the specific case of angiopoietin-2 and TNF*α*, the overexpression in PsV was significantly stronger than in PsA. CBD application to keratinocytes partially restored levels of MMP-1/2/3/7 and TIMP-1/2 (in an effect which was particularly enhanced by UVB irradiation), as well as levels of the examined angiogenic factors except TNF*α* (levels of which were increased in psoriatic keratinocytes and decreased in healthy keratinocytes). Presented results indicate that CBD may be suggested as an antiangiogenic factor that reduces the proinflammatory action of UVB in psoriatic keratinocytes and partially has a protective effect for healthy keratinocytes.

## 1. Introduction

Psoriasis vulgaris (PsV) is one of the most common immune-mediated inflammatory diseases, which affects approximately 4% of the adult population [1]. Its symptoms are mainly related to excessive exfoliation of the epidermis; however, the underlying cause of the disease is related to dysfunction of the entire human immune system [2]. As a result, not only do metabolic disturbances occur in skin cells, changing its condition and appearance, but damage also occurs to internal structures such as joints, as observed in psoriatic arthritis (PsA) [2]. To date, a number of factors in the pathogenesis of psoriasis (stress, environmental pollution, xenobiotics, genetic factors) have been defined [3], and various metabolic pathways involved in the development of this disease have been described, including inflammatory and oxidative stress (and their metabolic consequences), leading to excessive stimulation of keratinocytes (KCs) for proliferation, as well as the formation of leukocyte infiltrates into the dermis and vascular angiogenesis [4, 5]. Considering the fact that psoriasis is an immune inflammatory disease associated with an increased level of proinflammatory factors, mainly at the site of damage (skin, joints), an important issue is to determine the role of the proteolytic/antiproteolytic balance resulting from the levels or activity of matrix metalloproteinases (MMPs) and their inhibitors (tissue inhibitors of MMPs: TIMPs) in the pathogenesis of psoriasis, with the precise determination of the molecular mechanisms of this disease and therapy.

Extracellular MMPs belong to a large family of multidomain zinc endopeptidases. They are one of the most important proteolytic enzyme families which degrade components of the extracellular matrix, maintaining their physiological level, which is particularly meaningful in the case of skin cell migration and wound-healing. Moreover, MMPs are involved in many physiological processes, such as apoptosis or angiogenesis, especially in the human skin exposed to UV rays [6]. Oxidative stress markers and oxidatively modified lipids, levels of which are elevated in psoriasis and after UV irradiation, increase the expression of certain MMP genes and/or these enzymes' activities, both in blood and in psoriatic skin lesions [7, 8]. In the case of psoriasis, MMP activation is key to the incidence of structural changes in the epidermis via the modification of intracellular contacts and the composition of the extracellular matrix and to the promotion of angiogenesis in the skin, which favors the infiltration of immune cells [9]. Moreover, in addition to metabolic changes, these enzymes are additionally stimulated by UV radiation commonly used in the most psoriatic skin phototherapies [10]. This increased MMP activity has also been linked to other pathologies, including tumor cell spreading (metastasis), arthritis, periodontal disease, cardiovascular disease, and neurodegenerative diseases [11]. To prevent the negative effects of the MMP overexpression in the body, levels of endogenous inhibitors of these enzymes (TIMPs) are tightly physiologically regulated, as is the MMP:TIMP ratio. These balances may be dysregulated under pathological conditions [12].

Research is underway to identify compounds, including natural ones that would aid treatment. So far, the natural phytocannabinoid cannabidiol (CBD) has proved to be a promising candidate, especially for use in pharmacotherapy combined with UV phototherapy [13]. CBD is characterized by its antioxidant, as well as anti-inflammatory properties, consisting of support for endocannabinoids in their interaction with specific transmembrane receptors, such as cannabinoid receptors and TRPVs (transient receptor potential channels—vanilloid subtype) or PPARs (peroxisome proliferator-activated receptors) [14]. These features allow the determination of CBD as a cytoprotective compound, especially in relation to UV-irradiated skin cells [15]. Moreover, CBD has been found to reduce MMP secretion and activity, as well as upregulation of MMP inhibitors such as TIMP-1 in various types of cancer development [16, 17]. This inhibitory effect of CBD on MMPs was also observed in unchanged skin fibroblasts [18]. So far, CBD is used extensively in skincare products to avoid inflammation, dryness, and the generation of free radicals in irritated tissue [19]. However, its protective properties are constantly being considered in the design of new therapeutic applications [20].

Therefore, the aim of this study was as follows: firstly, to analyze how the development of PsV and PsA affect the expression of basic MMPs and their inhibitors, as well as growth/angiogenesis factors in plasma, and secondly, to determine the effect of CBD on these parameters in KCs isolated from psoriatic patients and irradiated in vitro with UVB, simulating the conditions experienced under phototherapy.

## 2. Materials and Methods

### 2.1. Material

#### 2.1.1. Plasma Sample Collection

Plasma samples were obtained from the blood of 20 patients with psoriasis vulgaris (12 females and 8 men, mean age: 37) and from 20 people with diagnosed psoriatic arthritis (9 females and 11 men, mean age: 36). The control group was composed of 10 healthy females and 10 healthy men (in total: 20, mean age: 36). All psoriasis vulgaris patients were chosen on the basis of having a diagnosis of plaque psoriasis for at least 6 months prior, with at least 10% of the total body surface area affected. The severity of psoriasis was assessed using the Psoriasis Area and Severity Index (PASI) score (median 21; range 15–25). Patients with psoriatic arthritis were diagnosed on the basis of a questionnaire CASPAR (Classification Criteria for Psoriatic Arthritis). None of the respondents received topical, oral, or injectable medications during the 4 weeks before the study, and none had comorbidities, had been a smoker, or had abused alcohol. The presence of other both skin as well as systemic diseases excluded patients from the study. The study was conducted in accordance with the Declaration of Helsinki, and the protocol for the collection of all (blood or skin) samples was approved by the Local Bioethics Committee in Medical University of Bialystok (Poland), No. R-I-002/289/2017. Written informed consent was obtained from all participants.

Blood was collected into EDTA tubes and, to prevent oxidation in obtained samples, butylhydroxytoluene (BHT) was used. Plasma was then obtained by spinning for 25 min in 300 × g. Isolated material was stored at -80°C before further analysis.

#### 2.1.2. Cell Line Derivation

KC cell lines were isolated from psoriatic skin biopsies of 5 randomly chosen patients with psoriasis vulgaris (2 men and 3 women; age range 25–48 years, mean 40). Biopsies were also taken from five healthy people (sex- and age-matched individuals forming a control group; age range 27–51 years, mean 41) who had moles, which were removed along with the adjacent skin. Skin biopsies were obtained from active psoriasis lesions from the elbow or knee area. The samples were collected using the classical surgical method. After skin decontamination, infiltration anesthesia with 1% lignocaine was applied, and then skin fragments of approximately 5-8 mm were collected. Samples immediately after biopsy were destined for histopathological examination (hematoxylin-eosin staining, data showed previously [21]). The remaining material was incubated overnight at 4°C in dispase (1 mg/mL) to separate epidermis from dermis. Following incubation, the epidermis, mainly composed of KCs, was digested using trypsin/EDTA in a 20 min incubation at 37°C. Separated KCs were washed in PBS and resuspended in growing medium: Keratinocyte Serum-Free Medium (Gibco, Grand Island, NY) supplemented with fetal bovine serum (10%), penicillin (50 U/ml), and streptomycin (50 *μ*g/ml). Cells were then cultured under standard conditions in a humidified atmosphere of 5% CO_2_ at 37°C. Cell culture purity and uniformity were controlled based on the KC morphology observation.

#### 2.1.3. Cell Treatment and Medium Collection

When KCs formed all derived lines reached 70% confluence, cells were exposed to UVB (312 nm) radiation in cold PBS (4°C) to avoid heat stress and oxidation of the medium components. Used wavelength corresponds to the wavelength of the UVB radiation (Narrov Band) used in the phototherapy of psoriasis [22]. KCs were irradiated on ice at a distance of 15 cm from an assembly of 6 UV lamps (Bio-Link Crosslinker BLX 312; Vilber Lourmat, Germany) at 6 W each, corresponding to a flux of 4.08 mW/cm^2^. Total UVB dose was 60 mJ/cm^2^, which resulted in the death of approximately 30% of KCs. After irradiation, KCs were incubated for 24 hours under standard conditions in medium without supplementation with growth factors. To analyze the effect of CBD on these cells, a suspension of CBD in ethanol was added to a final concentration of 4 *μ*M (the final concentration of ethanol was 0.3%). Following incubation (24 h), the medium from was harvested and frozen at -80°C before further analysis.

### 2.2. Methods

#### 2.2.1. Metalloproteinase Activity

Activity of metalloproteinases MMP-2 and MMP-9 was measured using gelatin zymography [23]. Samples were electrophoretically separated on an 8% gel containing gelatin (2 mg/ml). MMP activity was induced by incubation of the gel for 20 hours in 37°C in Tris-HCl buffer (50 mM, pH 7.8) with CaCl_2_ (5 mM) and Triton X-100 (0.1%). Following incubation, the gel was stained in Coomassie Blue. Next, densitometric analysis of light fringes on a dark background was carried out using a Versa Doc System and Quantity One software (Bio-Rad Laboratories Inc., CA, USA).

#### 2.2.2. Protein Expression

**Figure 1:**
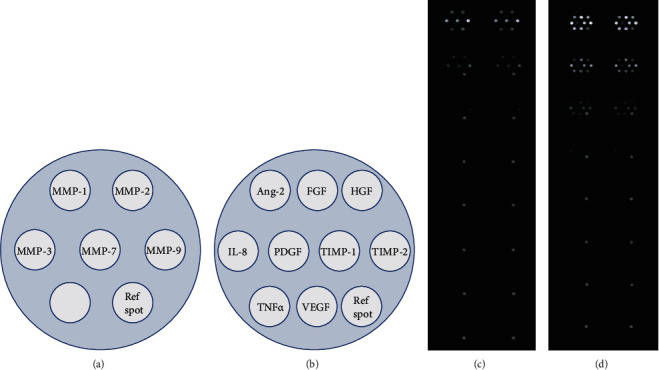
The spot distribution in the Q-Plex™ Human MMP (a) and Q-Plex™ Human Angiogenesis (b) and the images of the obtained curves for targeted proteins (c, d).

Levels of metalloproteinases MMP-1, MMP-2, MMP-3, MMP-7, and MMP-9 and their inhibitors, TIMP-1 and TIMP-2, as well as growing factors/angiogenesis HGF, VEGF, PDFG, FGF, IL-8, angiopoietin-2, and TNF*α*, were measured using commercially available kits (Q-Plex™ Human MMP (6-Plex) no. 340949HU and Q-Plex™ Human Angiogenesis (9-Plex) no. 150249HU, Quansys Biosciences, UT, USA) [24]. These assays use a quantitative chemiluminescent method of detection based on an ELISA protocol. These assays used two different antibodies specific for their respective targets. Samples, as well as calibrators, were pipetted into wells of a 96-well plate arrayed with analyte specific anti-bodies that captured chosen protein within 1 hour. Specific proteins were immobilized to their locations in the array. After washing away any unbound protein, a mixture that contains biotinylated analyte specific antibodies was added and incubated for 1 hour. The biotinylated antibodies completed the sandwich for each specific arrayed analyte. After washing away unbound biotinylated antibody, streptavidin-horseradish peroxidase (SHRP) was added for 20 min. Following an additional wash, the signal reading was performed by Q-ViewTM Imager LS with Q-View Sotware (Quansys Biosciences, Logan, UT, USA) according to the manufacturer protocols and method improvements available online at https://www.quansysbio.com/support/tech-tips/. Protein levels were read according to the respective calibration curves in the range: 49.38–12,000 pg/ml (MMP-1), 329.22–80,000 pg/ml (MMP-2), 18.52–4500 pg/ml (MMP-3), 7.41–1800 pg/ml (MMP-7), 181.07–44,000 pg/ml (MMP-9), 13.7–10,000 pg/ml (Ang-2), 6.9–5000 pg/ml (FGF), 19.2–14,000 pg/ml (HGF), 5.5–4000 pg/ml (PDGF), 13.7–10,000 pg/ml (TIMP-1), 27.4–20,000 pg/ml (TIMP-2), 5.5–4000 pg/ml (TNF*α*), 2.7–2000 pg/ml (VEGF), and 2.7–2000 pg/ml (IL-8). The image of the obtained curves, as well as the distribution of measurement spots, is presented in Figure 1.

All results were normalized against total protein concentration, as measured by the Bradford assay [25].

### 2.3. Statistical Analysis

Data were analyzed using standard statistical analyses, including multivariate analysis (one-way ANOVA). The Shapiro-Wilk and the Leven tests were used to check the normality of the data distribution and the homogeneity of variance. Results are expressed as the mean ± standard deviation (SD).

## 3. Results

**Figure 2:**
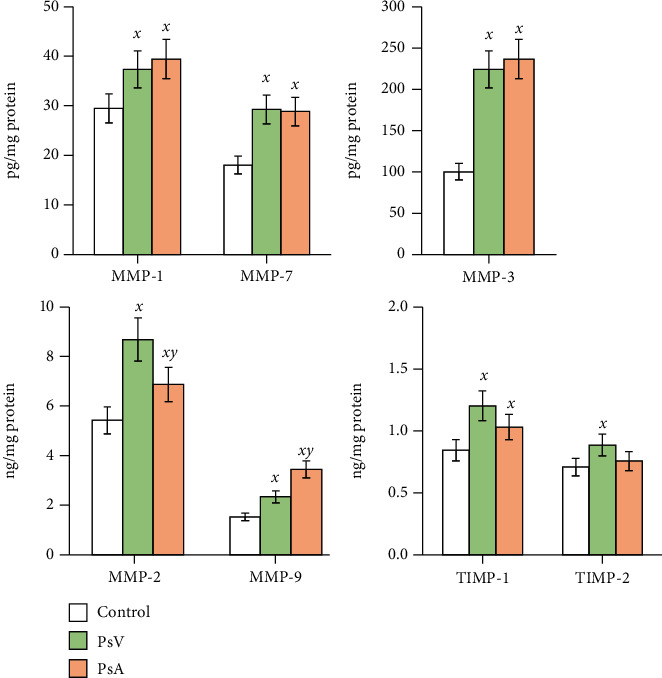
The level of metalloproteinases (MMP-1, MMP-2, MMP-3, MMP-7, and MMP-9) and metalloproteinase inhibitors (TIMP-1/2) in plasma of patients with psoriasis vulgaris (PsV, *n* = 20) and psoriatic arthritis (PsA, *n* = 20) compared to plasma of healthy subjects (control group, *n* = 20). Mean values ± SD are presented; ^***x***^ statistically significant differences vs. control group, *p* < 0.05; ^***y***^ statistically significant differences vs. PsA group, *p* < 0.05.

**Figure 3:**
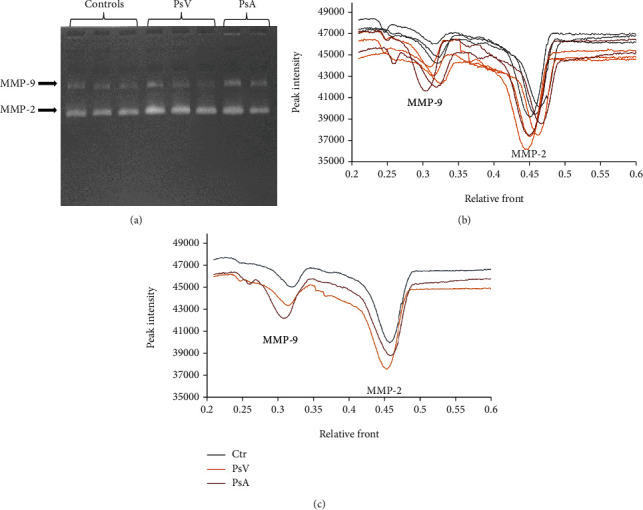
The activity of metalloproteinases (MMP-2 and MMP-9) in plasma of patients with psoriasis vulgaris and psoriatic arthritis compared to plasma of healthy subjects (control group). Presented results are (a) image of gelatin zymography, (b) the intensity of the signals for individual lines, and (c) the average of data from each group.

The metabolic changes resulting from the development of psoriasis are inextricably linked to the increased plasma levels/activity of MMPs in patients diagnosed with both PsV and PsA. Our results confirm this finding, especially in the case of MMP-1 and MMP-7, where an increase of 25–60% was observed in the plasma of PsV and PsA patients compared to the level of these MMPs in the control group (Figure 2). Moreover, the plasma level of MMP-3 in psoriasis patients (both PsV and PsA) was more than twice as high as in the control group. Also, the levels of MMP-2 and MMP-9 were increased in psoriatic plasma, with significant differences observed in different forms of the disease: MMP-2 levels were higher than in plasma from healthy subjects by about 60% in PsV and by 25% in PsA, while MMP-3 levels increased by 53% in PsV and by 125% in PsA (Figure 2). The differences in the levels of these enzymes also coincided with changes in their activities. MMP-2 from PsV patient plasma digested gelatin more strongly than this same enzyme from PsA patient plasma. An inverse relationship was observed for the bands etched by MMP-9 (Figure 3). The findings of increased MMPs level/activity in psoriasis plasma were accompanied with enhanced expression of tissue inhibitors of MMPs (TIMPs) (Figure 2). TIMP-1 level was increased in plasma of both PsV and PsA patients by 40% and 25%, respectively, while the TIMP-2 level was significantly increased (by 24%) only in plasma of PsV patients comparing to healthy subjects.

**Figure 4:**
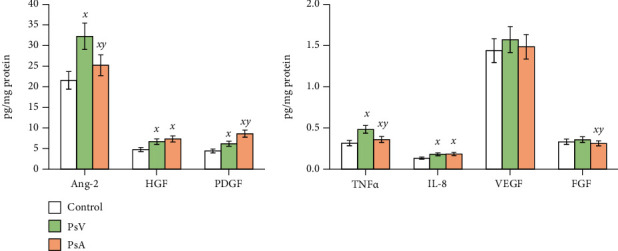
The level of the growth factors and markers of angiogenesis (angiopoietin-2, HGF, PDGF, TNF*α*, IL-8, VEGF, FGF) in plasma of patients with psoriasis vulgaris (PsV, *n* = 20) and psoriatic arthritis (PsA, *n* = 20) compared to plasma of healthy subjects (control group, *n* = 20). Mean values ± SD are presented. ^***x***^ statistically significant differences vs. control group, *p* < 0.05; ^***y***^ statistically significant differences vs. PsA group, *p* < 0.05.

Parallel to the disturbances in MMP activity in the plasma of psoriatic patients, significant changes were observed in the expression of the angiogenesis markers and growth factors in the plasma of both PsV and PsA patients (Figure 4). A higher increase in these transcripts was observed for angiopoietin-2 and TNF*α* in plasma of PsV patients, which was 50% more than in the control. The same parameters were also increased in plasma of PsA patients and were around 15% higher comparing to healthy subjects. Further, the expression of IL-8, HGF, and PDGF was enhanced in psoriatic patient plasma by at least 35%. Across VEGF and FGF levels, no significant changes were observed compared to controls except for the FGF expression in PsA patient plasma, which was decreased by approximately 7%.

**Figure 5:**
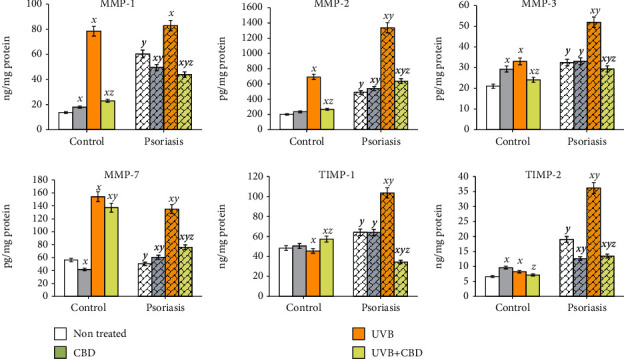
The level of metalloproteinases (MMP-1, MMP-2, MMP-3, and MMP-7) and metalloproteinase inhibitors (TIMP-1/2) in medium of keratinocytes isolated from psoriatic patents (*n* = 5) or healthy subjects (*n* = 5) and cultured following UVB irradiation (60 mJ/cm^2^) for 24 h with or without cannabidiol (CBD, 4 *μ*M). Mean values ± SD are presented; ^***x***^ statistically significant differences vs. non treated group (control or psoriatic, respectively), *p* < 0.05; ^***y***^ statistically significant differences between psoriatic and respective-treated control group, *p* < 0.05; ^***z***^ statistically significant differences between UVB + CBD and only UVB-treated group, *p* < 0.05.

Observed changes in the plasma of psoriatic patients were also reflected in the levels studied in isolated skin cells cultured in vitro. In the medium collected from psoriatic KCs, levels of MMPs (MMP-1/2/3) were significantly higher than in medium from parallel cultured KCs isolated from healthy individuals (increases of 4.4 times, 2.4 times, and 1.5 times, respectively) (Figure 4). Further, UVB radiation significantly enhanced MMP levels in medium of cultured psoriatic or control cells. However, this increase was greater in control cells than in psoriatic cells, e.g., the MMP-1 level in control cells following UVB exhibited a 5.7-fold increase, while in psoriatic KCs, this increase was only 1.4-fold. For all assessed MMPs (MMP-1/2/3/7), CBD caused significant reduction of their level in UVB-treated KCs. However, MMP-3 and MMP-7 levels were decreased by CBD more in UVB irradiated psoriatic KCs than in UV-irradiated healthy cells, while the MMP-1 and MMP-2 expression was reduced by CBD significantly more in UV-irradiated healthy cells as compared with the reaction of psoriatic KCs (Figure 4). Moreover, the TIMP expression in the medium of KCs was enhanced in psoriatic cells by compared to healthy cells by 32% for TIMP-1 and by 187% for TIMP-2. However, treatment of healthy cells with UVB radiation or CBD caused only moderate changes in the TIMP expression, while psoriatic KCs responded in a more significant manner. It was observed that UVB radiation caused an increase of 60% for TIPM-1 and 90% for TIMP-2 in psoriatic cells, while CBD significantly reduced the UVB-induced expression of these molecules by approximately 65–70% (Figure 5).

**Figure 6:**
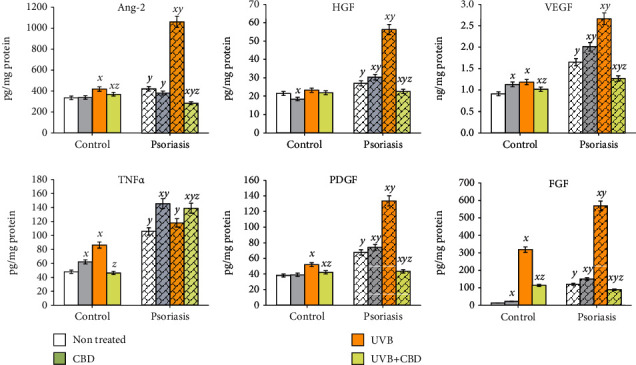
The level of growing factors and markers of angiogenesis (angiopoietin-2, TNF*α*, HGF, VEGF, PDGF, FGF) in medium of keratinocytes isolated from psoriatic patents (*n* = 5) or healthy subjects (*n* = 5) and cultured following UVB irradiation (60 mJ/cm^2^) for 24 h with or without cannabidiol (CBD, 4 *μ*M). Mean values ± SD are presented. ^***x***^ statistically significant differences vs. non-treated group (control or psoriatic, respectively), *p* < 0.05; ^***y***^ statistically significant differences between psoriatic and respective-treated control group, *p* < 0.05; ^***z***^ statistically significant differences between UVB + CBD and only UVB-treated group, *p* < 0.05.

Psoriatic changes in skin KCs were also related to the level of released into the medium growing factors and angiogenesis markers (Figure 6). Psoriasis development was associated with an increase in the level of angiopoietin-2 and HGF by 25%, of PDGF by 80%, of TNF*α* and VEGF twice, and of FGF three times as compared to healthy cells. Moreover, psoriatic KCs were also more sensitive to the experimental treatments, e.g., the expression of HGF in healthy cells was unwavering due to UVB radiation and CBD supplementation, while in psoriatic KCs, UVB induced double increase, which was prevented by CBD treatment. A similar character of changes following cell treatment was observed in the case of angiopoietin-2, PDGF, and VEGF, wherein UVB radiation resulted in a maximum of 30% increase in the expression in control cells and a 2-to-3-fold increase in psoriatic cells. Further, CBD treatment of these cells following UVB irradiation caused a decrease in these protein levels by approximately 15% in healthy KCs and 50–80% in psoriatic cells. The strongest effect of UVB and CBD action was visible in the changes of the FGF expression, where UVB caused a 3- and 5-fold increase in healthy and psoriatic cells, respectively, and CBD prevented these changes by 65% and 85%, respectively. CBD did not decrease only the level of proinflammatory factor TNF*α*, which is also strongly increased in psoriatic cells with and without UVB radiation, as well as following CBD treatment. On the other hand, CBD reduced the level of TNF*α* to the control level in KCs obtained from healthy people and treated with UVB (Figure 6).

## 4. Discussion

The healthy appearance of the human skin has always been seen as an indicator of a healthy body condition. Therefore, hyperexfoliation of the epidermis caused by chronic inflammation in patients with psoriasis vulgaris (PsV) is not only a dermocosmetic problem but may indicate serious pathological changes in the body, even leading to the development of other diseases, such as metabolic syndrome or Leśniowski's disease (Crohn's disease), or may cause psychological/psychiatric disorders [26]. A comorbid disease classically associated with PsV is psoriatic arthritis (PsA). It is not known what factors lead to the development of PsA in some cases of PsV [2], but many reports indicate that changes in the metabolic profile and activity of plasma proteins/blood cells differ significantly in both diseases [27, 28], which indicates the need for careful analysis to determine the most effective treatments in each case.

All significant changes occurred in cellular metabolism are usually reflected in the body fluids protein profile. This also is visible in the case of psoriasis, where changes in skin cell metabolism are reflected in plasma composition [29]. So far, the reorganization of the psoriatic epidermis connected with protease and peptidase stimulation has been observed also as the increased level of these proteins in patients' plasma [30]. Among these, MMPs can also be mentioned [9]. The major functions of MMPs are remodeling and degradation of extra-cellular matrix and cell membranes during various biological processes, such as cell migration and KC proliferation, as well as angiogenesis. There are five subfamilies of MMPs: collagenases, gelatinases, stromelysins, matrilysins, and membrane-type MMP; however, all are involved in the degradation of collagens, proteoglycans, and various glycoproteins. Their stimulation or repression might be regulated at the level of biosynthesis, while their activity can be directly modified by growth factors, cytokines, or tissue inhibitors of MMPs (TIMPs) [12].

It has been known for many years that psoriasis is associated with increased activity of various types of MMPs [9]; however, presented in this study are results which for the first time clearly indicate that in the case of plasma MMPs with collagenase or stromelysin activity, there are no differences between PsV and PsA patients, while gelatinases such as MMP-2 and MMP-9 significantly differentiate these two types of psoriasis. The plasma level as well as the activity of the MMP-9 in PsA is higher than in PsV, which makes the symptoms of this disease closer to that of rheumatoid arthritis (RA) [31]. However, in RA, MMP-2 is present only in latent form [32], while in PsA as in PsV, both level and activity are significantly increased. Moreover, these results are accompanied with increased level of MMP inhibitors TIMP-1 and -2. TIMPs act by binding to MMPs prevent their catalytic activity which, in the case of psoriasis, could limit inflammatory response and the scope of epidermal changes. Therefore, in the body's protective response, an increase in TIMP levels has been found in psoriatic patients' plasma [8]. The increased expression of TIMP-2 in PsV patient plasma also differentiates them from patients with PsA, where this was not observed. The inhibitory effect of TIMP-2-specific inhibition on MMP-2 (previous findings show a strong balance between MMP-2 and TIMP-2 in the case of cancer cells [33]) can explain the observed lack of TIMP-2 overexpression in PsA patient plasma.

These findings, showing an increase in MMP activity in psoriatic patient plasma, are also accompanied by increased markers of angiogenesis. New blood vessel formation in PsV patients favors Th1 and Th17 lymphocyte migration, as well as distribution of KC proliferation stimulators including growth factors, which additionally accelerates the pathogenesis of psoriasis [34]. However, one previous study suggests that abnormalities of vessel architecture and circulating levels of angiogenic growth factors have been observed in both psoriasis and atherosclerosis [35]. In the present study, most of the analyzed markers of angiogenesis in psoriatic patient plasma are similarly upregulated in both PsV and PsA; however, in the case of angiopoietin-2 and TNF*α*, the overexpression in PsV is significantly stronger than in PsA. Angiopoietin-2 (which is responsible for activation of the Tie-2 receptor) destabilizes blood vessel maturation, preparing them for new sprout formation and stronger psoriatic lesson invasion [36]. Additionally, it has been found that angiopoietin-2 is upregulated by VEGF and FGF [37], which were found to be elevated in PsV patients. Moreover, similar data for the TNF*α* expression differentiating PsV and PsA have been observed previously in psoriatic granulocytes [5], suggesting weaker proinflammatory signaling in PsA than in PsV, connected with stimulation or silencing by the protective mechanisms of the joints. Therefore, the inflammatory processes might be stronger in patients with PsV than in PsA. As a result, the mild variant of PsV is 10 times more frequently efficiently treated than severe form of psoriasis including PsA [38].

When analyzing the pathogenesis of psoriasis vulgaris, it is difficult to state unequivocally whether bothersome skin changes are the result of changes in the level of plasma signaling molecules, including proinflammatory cytokines, factors stimulating KC proliferation, as well as angiogenesis, or whether changes in the level of these factors in the plasma result directly from changes in epidermal cell metabolism [3]. This problem results from the complexity of the pathogenesis of psoriasis, the development of which consists of both environmental and genetic factors [4]. Regardless of this, skin cells are in constant communication with the immune cells present in the plasma and the signaling factors produced by them. Therefore, changes in plasma are reflected by changes in psoriatic KC metabolism and vice versa [21]. The increase in MMPs and growing factor level in plasma observed in this study from psoriatic patients may be the result of KC hyperproliferation, as well as a stimulus for the further development of the disease. Similar MMP level increase in psoriatic skin cells has been previously observed and has been associated with the induction of their expansion by IL-17 released from activated T lymphocytes [7].

All changes described in plasma markers indicating disturbances in organism homeostasis may have a detrimental effect on normal psoriatic patient functioning; therefore, this study proposes CBD as a potential compound that will decrease negative symptoms of psoriasis observed in skin KCs, especially after their UVB irradiation, received during phototherapy. CBD, because of its lipophilic nature, is wildly used in skincare products to avoid dryness of the skin [19]. Moreover, its antioxidant and anti-inflammatory properties provide protection of both healthy or psoriatic skin cells against oxidative stress caused by UV radiation [13–15, 39]. The effect of CBD on the level of MMPs in UVB irradiated KCs has not been studied so far; however, the results of studies from cancer cells indicate an inhibitory effect of CBD on MMPs, which is important in preventing the development of cancer [16, 17]. The results of this study show that CBD significantly decreases only MMP-7 level in healthy cells; however, it shows strong inhibitory effect for MMP-1/2/3/7 in UV-irradiated KCs, especially in the case of psoriatic cells. Other studies also show that CBD has the strongest inhibitory effect on MMPs in skin cells exposed to proinflammatory factors, e.g., TNF*α* [40], which is additionally increased in psoriatic KCs after UVB irradiation. Similar effects of CBD under inflammatory conditions connected with rheumatoid arthritis development are observed in synovial fibroblasts, where CBD decreases MMP-3 level by activating transient receptor potential ankyrin (TRPA1), and increasing intracellular calcium levels [41].

On the other hand, CBD as well as UVB irradiation in various ways affects the level of MMP inhibitors (TIMPs) in both control and psoriatic KCs. While in healthy cells, UVB irradiation and CBD treatment (separately or together) only slightly influence the levels of TIPM-1/2; in psoriatic cells, a strong increase is observed in TIMP level following UVB irradiation. Psoriatic skin cells are continuously stimulated to proliferation; therefore, their metabolism is still maintained at a high level, and thus the observed reaction is significant compared to healthy cells. Moreover, this may be a result of the protective role of TIMPs against UVB-induced degradation of collagen and elastic fibers [42]. Therefore, a CBD-induced decrease in TIMP levels following UVB radiation could potentially be of great importance in phototherapy of psoriasis. To date, many findings show that CBD increases TIMP levels primarily in cancer cells, which, via MMP inhibition, prevents cancer cell migration [17, 43, 44]. In the case of psoriatic skin cells, the effect is the opposite, similarly to umbilical vein endothelial cells [45]. This may be connected with other TIMP actions in KCs, including growth and apoptosis induction, as well as angiogenesis regulation [46], which require special regulation during the hyperproliferation of epidermal cells in psoriatic patients.

As mentioned, angiogenesis plays an important role in psoriasis development, and compared to healthy cells, psoriatic KCs are much more responsive to UVB radiation by overproducing growth factors and angiogenesis markers. However, in most cases, CBD significantly decreases these proteins' expression, which can prevent UVB-induced angiogenesis. Moreover, the natural stimulation of cell proliferation after UVB radiation is observed as the increased expression of HGF, VEGF, PDGF, or FGF only in psoriatic cells and is inhibited by CBD. Similar CBD effects have been observed in the case of VEGF or FGF in human prostate or colon cancer with enhanced cell proliferation [40, 47], similar to that seen in psoriasis. This suggests that CBD may support UVB phototherapy of psoriasis by limiting proliferative signaling in irradiated cells.

The results of this study indicate that CBD regulates the proteolytic/antiproteolytic balance in relation to the extracellular matrix, both in healthy and psoriatic cells. However, in the case of the proinflammatory factor TNF*α*, CBD significantly differentiates the direction of changes taking place in healthy and psoriatic KCs exposed to UVB radiation. It has long been known that TNF*α*, as a signaling molecule, not only induces a proinflammatory response of cells but is also a factor actively promoting the development of psoriasis [48]. Moreover, the TNF*α* expression is stimulated in skin KCs also in response to UVB radiation [49]. However, the antioxidant and anti-inflammatory properties of CBD contribute to the prevention of TNF*α* generation in control cells, what demonstrates the protective effect of this phytocannabinoid [50]. Simultaneously, CBD induces the proinflammatory action of UVB in relation to psoriatic cells by enhancing the TNF*α* level. However TNF*α* has been shown to inhibit the following: the secretion by plasmacytoid dendritic cells (pDC) of interferon gamma (IFN*γ*—an immunoregulatory cytokine that induces the release of inflammatory cytokines), the proliferation of KCs, and the maintenance of psoriasis [51]. Therefore, taking this into account, it can be suggested that CBD enhances the effects of narrowband UVB phototherapy.

## 5. Conclusion

In this study, it was shown that increased plasma levels of MMP-9 and TIMP-2, as well as angiogenic growth factors (mainly Ang-2), differentiate PsV from PsA. At the same time, these increased plasma levels are accompanied by a significant increase in the transcription factor TNF*α*. This indicates a significant exacerbation of metabolic/pathological changes in PsV patients. Therefore, the application of CBD with antioxidant and anti-inflammatory properties to UVB irradiated KCs has shown very significant effects. CBD has been found to prevent the disruption of the proteolytic/antiproteolytic balance established by extracellular matrix proteases and their inhibitors in both control and psoriatic KCs, especially in those exposed to UVB radiation. Moreover, on the basis of the obtained results, CBD can be indicated as an antiangiogenic factor, significantly reducing the level of all assessed angiogenic growth factors. At the same time, the anti-inflammatory effect of CBD is manifested by a decrease in the level of TNF*α* in KCs of the healthy skin, but an increase in the level of this transcription factor in psoriatic KCs, especially those exposed to UVB rays. Thus, CBD may prove to be the drug with beneficial effects on both healthy and psoriatic KCs, which may have important clinical implications.

## Data Availability

The data used to support the findings of this study are included within the article.
